# Synthesis, characterization and antimicrobial properties of two derivatives of pyrrolidine-2,5-dione fused at positions-3,4 to a dibenzobarrelene backbone

**DOI:** 10.1186/s13065-022-00801-5

**Published:** 2022-03-03

**Authors:** Emmanuel Sopbué Fondjo, Abdou Salamou Njoya, Jean-de-Dieu Tamokou, Giscard Doungmo, Bruno Ndjakou Lenta, Peter F. W. Simon, Apollinaire Tsopmo, Jules-Roger Kuiate

**Affiliations:** 1 Laboratory of Applied Synthetic Organic Chemistry, Department of Chemistry, Faculty of Science, University of Dschang, P.O. Box 67, Dschang, Republic of Cameroon; 2 Research Unit of Microbiology and Antimicrobial Substances, Department of Biochemistry, Faculty of Science, University of Dschang, PO Box 067, Dschang, Republic of Cameroon; 3 Institut Für Anorganische Chemie, Christian-Albrechts-Universität Zu Kiel, Max-Eyth-Straße 2, 24118 Kiel, Germany; 4 Higher Teacher’s Training College, University of Yaounde I, P. O. Box 47, Yaounde, Cameroon; 5 Polymer Chemistry Laboratory, Faculty of Live Sciences, Rhein-Waal University of Applied Sciences, Marie-Curie Straße 1, 47533 Kleve, Germany; 6 Department of Chemistry, Carleton University, 1125 Colonel By Drive, Ottawa, K1S5B6 Canada

**Keywords:** Pyrrolidine, *N-*arylsuccinimid, Dibenzobarrelene, Phenols, Azo compound, Anti-microbial activities

## Abstract

A new diazo derivative of a pyrrolidine-2,5-dione (**8**) fused at position-3,4 to a dibenzobarrelene backbone has been prepared by coupling the previously reported *N-*arylsuccinimid (**5**) precursor with aryldiazonium ion of aniline. The initial step of the reaction involved the preparation of the intermediate 9,10-dihydro-9,10-ethanoanthracene-11,12-dicarboxylic anhydride (**3**) through [4 + 2]-cycloaddition between anthracene and maleic anhydride in refluxing xylene which was then condensed with para-aminophenol to give compound **5**. Compounds **5** and **8** were characterized by their physical, elemental, and spectroscopic data. 2D-NMR (COSY, HSQC, and HMBC) techniques were used to confirm the structure of compound **5**. Compounds **5** (MIC = 32–128 μg/mL) and **8** (MIC = 16–256 μg/mL) along with the precursor **3** (MIC = 64–128 μg/mL) displayed moderate to low antimicrobial activities against selected bacterial and fungal species when compared with those of nystatin (MIC = 0.50–2 μg/mL) and ciprofloxacin (MIC = 0.50–16 μg/mL) used as reference drugs.

**Graphical Abstract:**

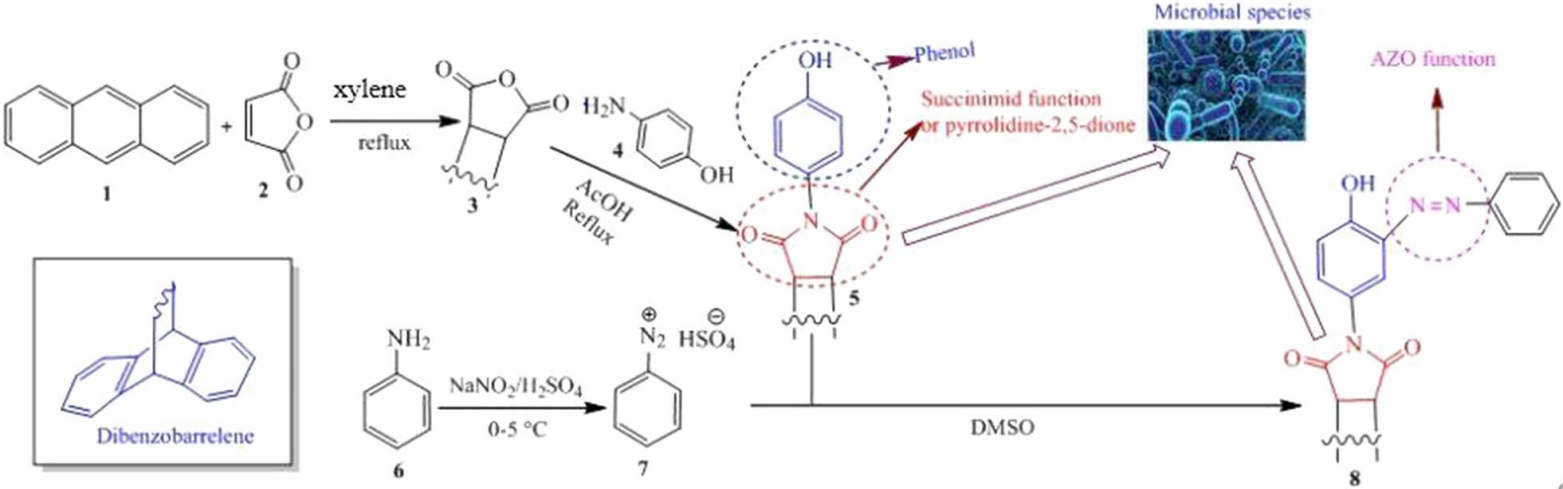

**Supplementary Information:**

The online version contains supplementary material available at 10.1186/s13065-022-00801-5.

## Introduction

**Scheme 1:**
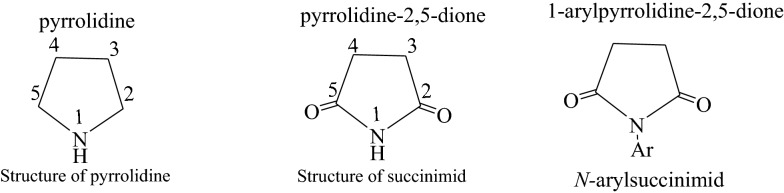
Structure of pyrrolidine and their derivatives

Pyrrolidines, also known as azolidines, are the simplest compounds in the azolidine group. They are cyclic amines with four carbon atoms having the general formula C_4_H_9_N. Pyrrolidine derivatives known as succinimid are cyclic imides with five vertices (Scheme 1) [1]; the simplest compound of this family is the succinimid of formula C_4_H_5_NO_2_. The substitution of the nitrogen proton with aromatic groups yields *N-*arylsuccinimid type compounds. Pyrrolidines and their derivatives are essential structural units of many important compounds useful in the pharmaceutical field because they possess biological functions such as antimicrobial [2, 3], antitumor [4], anticonvulsant [5], antitubercular [6], and analgesic activities [7].

Synthetic compounds containing azo moieties have been found to possess biological functions similar in some cases to those of *N-*arylsuccinimid (e.g. antimicrobial [8, 9], antiinflammation [10], antioxidant [11]). In addition to these two groups biologically active compounds of synthetic origin, many natural occurring compounds such as phenols and polyphenols are biologically active. They are being extensively studied in various models and some their activities include antioxidants but also anti-tumor, antiinflammatory and antimicrobial [12–14].

**Scheme 2:**
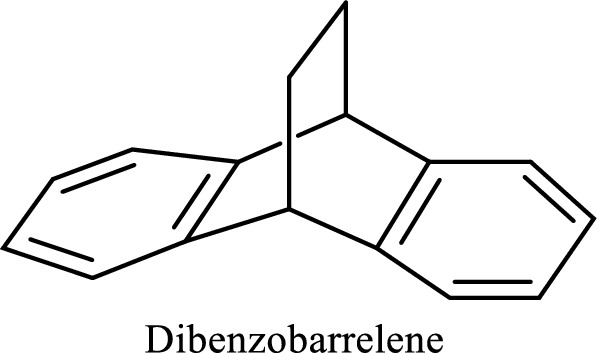
Basic structure of dibenzobarrelene

Antibiotics have been widely used in the past decade to treat a variety of infectious diseases that remain one of the leading causes of mortality and morbidity in the world. Nevertheless, the massive use of these antibiotics has led to the emergence of pathogens multi resistant to conventional antibiotics [15]. Such resistant pathogens include the case of methicillin resistant *Staphylococcus aureus*, vancomycin resistant *Enterococcus*; which sets the limits of the therapeutic treatments currently used [16]. One of the possible ways to fight this phenomenon is the development of new molecules. Previous work on pyrrolidine derivatives and azo compounds show that these compounds are very important because of their multiple biological activities [17–22]. Furthermore, Mkpenie and co-workers [23] recently found that the azo moiety (–N = N–) was a pharmacophore responsible for activities in azo compounds. The motivation of this work is that to the best of our knowledge, azo compounds having the nucleus of dibenzobarrelene (Scheme 2) [24] have hitherto not been reported in the literature.

Furthermore, in contrary to pyrolidines, phenols and azo compounds, very few is known about the biological activity of dibenzobarrelene derivatives. Despite the individual biological function of *N-*arylsuccinimid, azo compounds and phenol molecules, synthesis strategies to incorporate them into a single molecule may be advantageous. That's why we combined in this work in a single molecular architecture the pyrrolidine-2,5-dione, phenol, fragment of dibenzobarrelene and the azo bridge, with the expectation to obtain a hybrid molecule with improved biological potentials.

## Results and discussion

### Chemistry

**Scheme 3:**
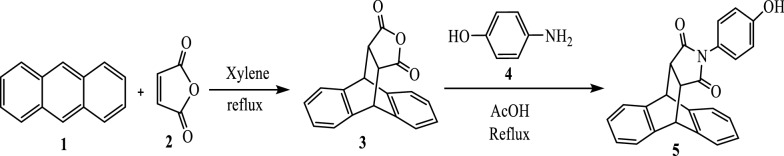
Reactions sequences to compound **5**

The preparation of compound **5** was done by the following procedure: A Diels–Alder reaction between anthracene **1** and maleic anhydride **2** leads to compound **3** which was subsequently condensed with para-aminophenol **4** in acetic acid at reflux to give the desired compound **5** with yield of 92% (scheme 3).

UV–visible spectrum shows that this compound **5** absorbs between 200 and 400 nm, the near UV range. This spectrum has several absorption bands; λ_1max_ = 250 nm, λ_2max_ = 355 nm, λ_3max_ = 395 nm corresponding respectively to the electronic transitions π-π* of chromophores C = C of benzene present in the base of dibenzobarrelene, of C = O and C = C of benzene present in succinimid. The high value of λ_3max_ is explained by the presence of auxochrome OH on benzene.

Its IR spectrum shows a characteristic broad absorption band around 3363 cm^−1^ attributable to the OH function of phenol. At 2973 cm^−1^can also be observed a band corresponding to the valence = C–H bonds of the benzene ring; the absorption band at 1696 cm^−1^ is attributable to the carbonyls (C = O) of the amides. Those at 1600 cm^−1^ and 1562 cm^−1^ are attributable to the valence bonds C = C of the aromatic cycle. The C–N and C–O functions are characterized by the presence of the bands at 1273 cm^−1^ and at 1202 cm^−1^ respectively.

Its mass spectrum shows two peaks of pseudo molecular ions, one at 390 (100%) corresponding to [M + Na]^+^ and the other at 757 (90%) corresponding to [2 M + Na]^+^ from which the molar mass of the compound was deduced to be m/z: 367 corresponding to the raw formula C_24_H_17_NO_3_.

Its ^1^H NMR spectrum shows, despite the symmetry, that the aromatic protons in dibenzobarrelene moiety are not equivalent due to the molecular arrangements [25]; so they have different signals. The doublet split at 7.32 (dd, 2H, J = 5.3 and 3.3 Hz) is assigned to the protons H-3, H-7 while that at 7.23 (dd, 2H, J = 5.4 and 3.3 Hz) is assigned to the protons H-2, H-6. The multiplet at 7.11 is attributed to the protons H-1, H-4, H-5, H-8. In the phenolic moiety, we have an AA′BB′ proton system. The doublet at 6.62 (d, 2H, J = 8.8 Hz) is assigned to the proton H-2′, H-6′ and that at 6.17 (d, 2H, J = 8.8 Hz) is assigned to the protons H-3′, H-5′. The low values of the chemical shifts of these benzenic protons are due to the mesomeric effect on the one hand of the OH group which shields in *ortho* position the protons H-3′, H-5′ and on the other hand the mesomeric effect of the nitrogen contained in the pyrrolidine cycle which also *ortho* shields the protons H-2′, H-6′. The mesomeric effect of OH is greater than that of nitrogen, hence the strong shielding of the H-3′, H-5′ protons compared to those of H-2′, H-6′. The singlet at 4.75 (s, 2H) is assigned to the benzylic protons H-9, H-10 and the other benzylic protons H-11, H-15 gives a singlet at 3.25 (s, 2H). The benzylic protons H-9, H-10, H-11, H-15 was expected to give doublets, but rather give singlets due to the presence of a more electronegative phenyl group adjacent to each proton [25]. The COSY spectrum of this compound shows squares of correlation between the protons H-3 and H-4, H-2 and H-1, H-2′ and H-3′, H-9 and H-11. The ^13^C NMR spectrum of compound **5** has thirteen signals instead of twenty-four as in the molecular formula which confirm that there is symmetry in the molecule. Five signals are observed corresponding to quaternary carbons at 177.9 (C = O), 157.4 (C–OH), 141.2 (C = C), 138.8 (C = C) and 122.7 (C–N). There are also six signals attributable to protonated benzenic carbons at 127.6, 127.1, 126.8, 125.1, 124.3, 115.9 and two signals attributable to benzylic carbons at 46.9 and 45.8. The data of the spectra of this compound are in agreement with those found in the literature [26].

**Scheme 4:**
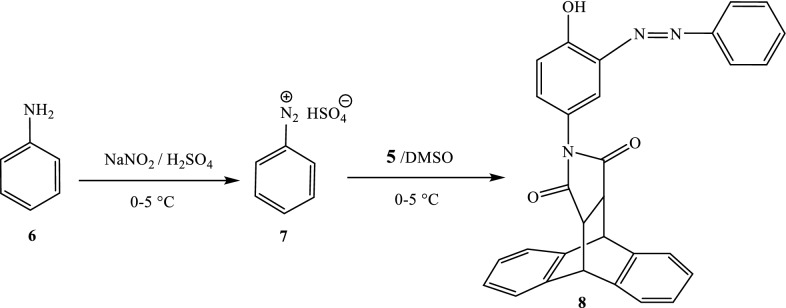
Reactions sequences to compound **8**

The synthesis of the azo compound was done in a two-step process including the diazotization of aniline (**6**) to form the diazonium ion **7** which then copulates with compound **5** to give the azo compound **8** with yield of 67% (Scheme 4).

The UV–visible spectrum of **8** showed a large band around λ_max_ = 385 nm corresponding to the electronic absorption of the chromophores of the system contain azo group. There is also an extension of the peak and an increase in the absorbance of this compound to more than 1.5 compared to that of the compound **5**; moreover, the conjugation of the C = C chromophores of arylsuccinimid and aniline by the azo bridge –N = N– promotes the absorption of this compound beyond 400 nm, in the visible region. In its IR spectrum, characteristic absorption bands for phenol and = C–H of the benzene ring are present at 3367 and 3060 cm^−1^, respectively. The absorption bands at 1768 cm^−1^ and at 1696 cm^−1^ are attributable to the carbonyls (C = O); the higher frequency band is allocated to symmetrical vibrations and the lowest frequency band is allocated to asymmetrical vibrations. The band at 1598 cm^−1^ is attributable to the valence bonds C = C of the aromatic cycle. The azo function (–N = N–) is confirmed by the presence of an absorption band at 1465 cm^−1^. The C–N and C–O functions are characterized by the presence of the bands at 1274 cm^−1^ and at 1202 cm^−1^ respectively. The absorption at 764 cm^−1^ is attributable to the deformation of (C–H) aromatic.

**Scheme 5:**
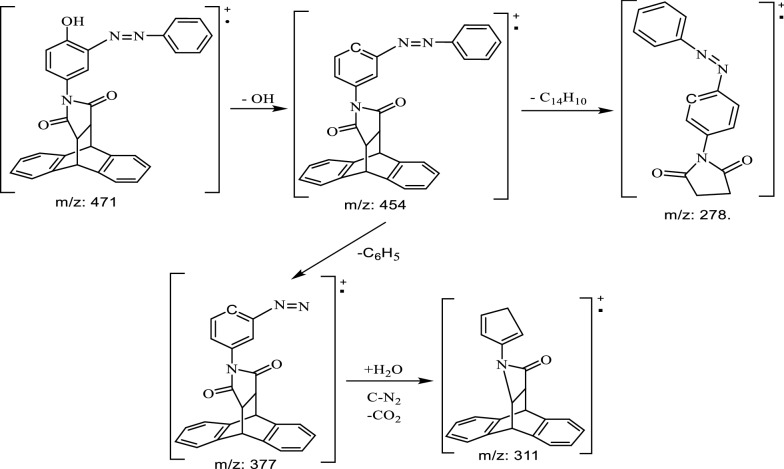
Some important fragments of compound **8** observed in the ESI^+^-mode MS

On its mass spectrum in ESI^+^ mode, we observed the pseudo molecular ions at 494 (100%) corresponding to [M + Na]^+^ from which the molar mass of the compound was deduced to be m/z: 471 corresponding to the gross formula C_30_H_21_N_3_O_3_ (Scheme 5). The mass spectrum of compound **8** also contained fragments ions at 454 (20%) [M^+^-OH], 394 (24%) [M^+^ -C_6_H_5_], 377 (18%) [M^+^-OH -C_6_H_5_], 311 (11%) [M^+^-OH-C_6_H_5_-CO_2_-C-N_2_ + H_2_O], 278 (15%) [M^+^-OH-C_14_H_10_].

On the ^1^H NMR spectrum of compound **8**, the protons of the aniline moiety H-2′′, H-3′′, H-4′′, H-5′′ and H-6′′ were exhibited at 7.45 (m, 5H) overlapping with the protons of the dibenzobarrelene nucleus. It’s also exhibited as in compound **5** the aromatic protons of the dibenzobarrelene moiety respectively at 7.40 (dd, 2H, J = 5.3 and 3.2 Hz), 7.32 (dd, 2H, J = 5.3 and 3.3 Hz), 7.19 (m, 4H). The benzylic protons appeared at 4.75 (s, 2H) and 3.25 (s, 2H). The phenolic moiety presents three types of protons, two doublets and a singlet. The singlet at 6.98 (s, 1H) is attributable to the proton H-6′ whereas, the doublets at 6.68 (d, 1H, J = 8.1 Hz) and 6.18 (d, 1H, J = 8.2 Hz) are respectively attributed to the protons H-2′ and H-3′. The ^13^C NMR of this compound showed more than sixteen carbons instead of thirteen as in compound **5**; confirming therefore the above suggested substitution pattern. In addition to all the carbons present in compound **5**, there are new carbon signals at 160.7 attributable to the depleted carbon C-1′′ of aniline carrying the azo group; that at 130.8 attributable to carbon C-5′ of the phenolic fragment bearing the azo group. Furthermore, one can notice an overlapping of the signals due to carbons C-2′′, C-3′′, C-4′′, C-5′′, C-6′′ at 129.1.

## XDR analysis

**Fig. 1:**
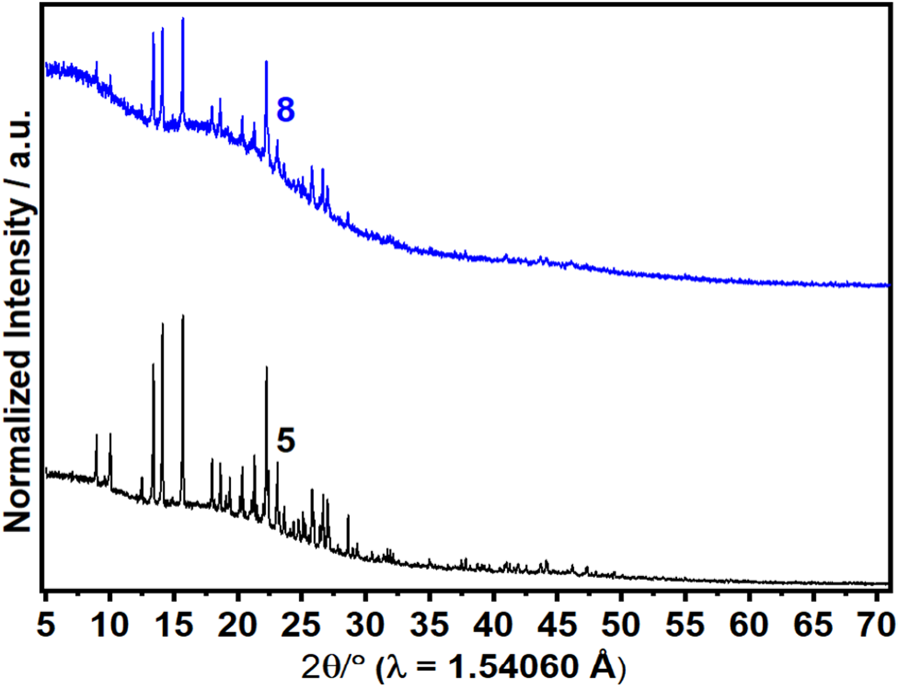
Ex situ PXRD pattern (Cu Kα1 radiation) of XRD of compound **5** and **8**

**Fig. 2:**
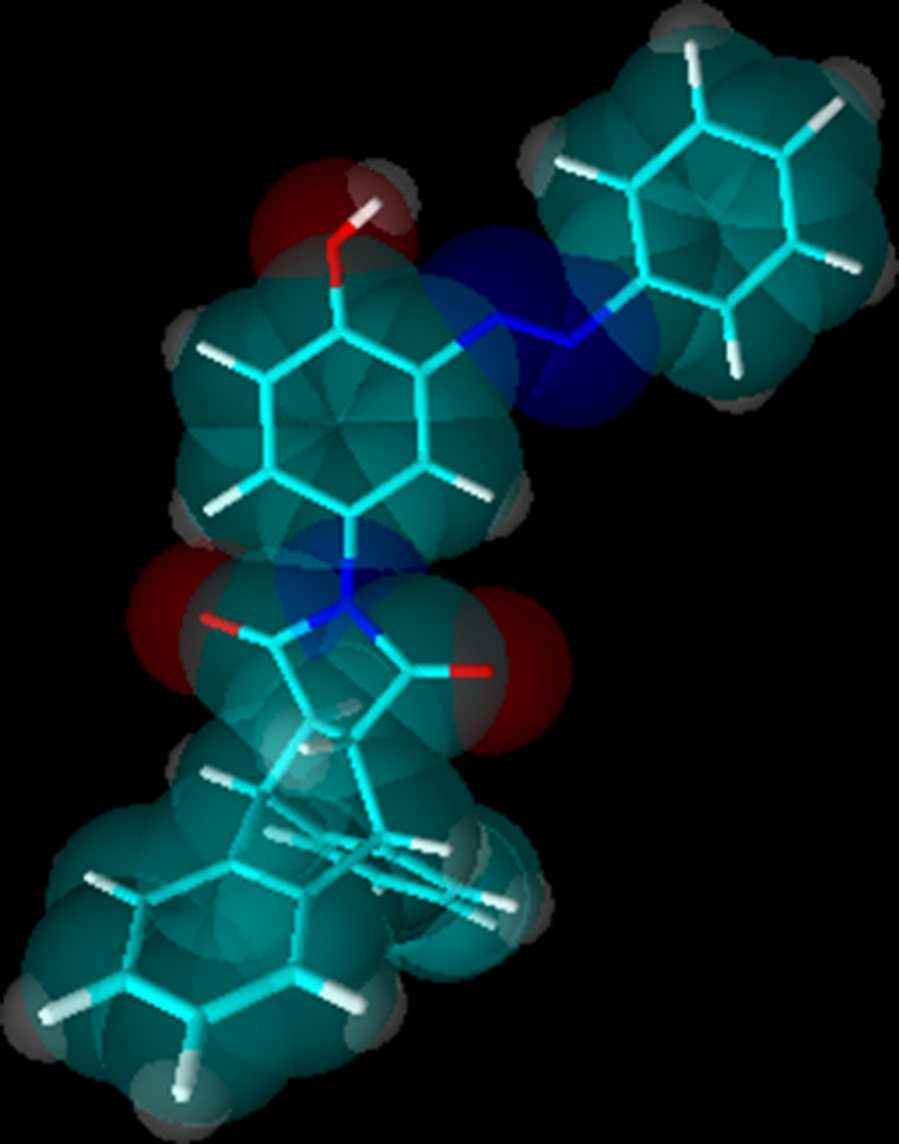
Optimized 3D view of compound **8**. The structure was drawn with the program ACD/3D viewer (freeware) of ACD/Labs

The spectra of the X-ray diffraction analysis of compounds **5** and **8** are different from each other (Fig. 1). A large number of intense bands or peaks is observed on the spectrum of compound **5**, whereas on the spectrum of compound **8** the number of bands is reduced and the intensities of the latter are low. This suggests that succinimid **5** has a better crystal structure and is therefore more stable than the azo compound [27]. In addition, this stability of compound **5** suggests a better cohesion between atoms compare to the azo compound [27]. This weak cohesion of atoms in the azo compound may be due to presence of the azo bridge (–N = N–). The optimized 3D view of compound **8** is clearly presented in Fig. 2.

## Biology

### Antimicrobial activity

The antimicrobial activities were evaluated on seven species of microorganisms including bacteria and yeasts and the data are summarized in Table 1. It appears from the results of these analyses that the activity of the compounds varies according to the nature of the microorganisms. Compounds **3**, **5**, **8** showed moderate to low antimicrobial activities respectively with MICs = (64–128) μg/mL, (32–128) μg/mL and (16–64) μg/mL on bacteria and 128 μg/mL, (64–128) μg/mL, (64–256) μg/mL on yeasts. It is noted that the introduction of the phenol fragment into compound **3** induced an increase in the activity of compound **5** in particular on *Staphylococcus aureus*, *Candida tropicalis* PK233, *Cryptococcus neoformans* H99 and remains unchanged on the other microorganisms. Furthermore, the introduction of the azo function in compound **5** resulted into an increase of the activity of compound **8**, in particular on *Staphylococcus aureus*, *Vibrio cholera* SG24, *Vibrio cholera* CO6. The yeasts have shown a low sensitivity with respect to the azo compound, thus showing that this compound has much better antibacterial than antifungal activity on these types of microorganisms.

**Table 1:** Antimicrobial activity (MIC, MBC and MFC in µg/mL) of synthesized compounds as well as reference antimicrobial drugs

Compounds	Inhibition parameters	*S. aureus*	*V. cholerae* NB2	*V. cholerae* SG24	*V* *cholerae* CO6	*C. albicans* ATCC10231	*C. tropicalis* PK233	*C. neoformans* H99
**8**	MIC	16	64	32	64	256	128	64
	MBC or MFC	32	128	64	> 256	> 256	> 256	128
	MBC or MFC /MIC	2	2	2	/	/	/	2
**5**	MIC	32	64	64	128	128	64	64
	MBC or MFC	32	128	128	> 256	> 256	> 256	> 256
	MBC or MFC /MIC	1	2	2	/	/	/	/
**3**	MIC	64	64	64	128	128	128	128
	MBC or MFC	128	128	128	> 256	> 256	256	256
	MBC or MFC /MIC	2	2	2	/	/	2	2
Reference drugs*	MIC	0.50	16	8	16	2	0.50	1
	MBC or MFC	0.50	16	8	16	2	0.50	1
	MBC or MFC /MIC	1	1	1	1	1	1	1

/ not determined, *MIC* Minimum Inhibitory Concentration, *MBC* Minimum Bactericidal Concentration, *MFC* Minimum Fungicidal Concentration

*Nystatin for yeasts and ciprofloxacin for bacteria were tested together with compounds **3**, **5** and **8**

All the compounds tested showed weak biological activities compared to the reference drugs. Bactericidal effects (MBC/CMI ≤ 4) were observed on *S. aureus*, *V. cholera* NB2 and *V. cholera* SG24 with compounds **8**, **5** and **3** while fungicide effect (MFC/CMI ≤ 4) was recorded on *C. neoformans* H99 with compounds **8** and **3** and on *C. tropicalis* PK233 with compound **3**. Compounds **8**, **5** and **3** showed bacteriostatic effect (MBC/CMI > 4) against *V. cholera* CO6 and fungistatic effect on *Candida albicans* ATCC10231. Compounds **8** and **5** displayed fungistatic effect (MFC/CMI > 4) on *C. tropicalis* PK233 while fungistatic effect was recorded on *C. neoformans* H99 with compound **3.**

The variations in the susceptibilities observed between the microorganisms and the compounds tested would be due to the differences in genetic constitutions that exist between the different microbial strains tested [28].

### Cytotoxic activity

The cytotoxic activity of azo compounds against red blood cells (RBCs) was investigated using Triton X-100 as a positive control. Interestingly, none of the tested compounds showed cytotoxic activity against RBCs at concentrations up to 256 μg/mL (results not shown). This finding supports the selective toxicity of the tested compounds towards the tested bacteria and fungi.

## Conclusion

The results of biological tests showed that compounds **3**, **5**, **8** possess antimicrobial activities. Although being less active than the compound taken as a reference, the azo compound has better antibacterial activity than the other two compounds especially on *Staphylococcus aureus*, *Vibrio. Cholera* SG24 and *Vibrio cholera* CO6 strains. The antimicrobial screenings revealed that all the tested compounds **8**, **5** and **3** have moderate to low antibacterial and antifungal activities. These results show that the azo function (N = N) is indeed a pharmacophore and would be responsible for the biological activity in the azo molecules.

## Materials and methods

### Instrumental method

All Melting points are corrected and were determined with a STUART SCIENTIFIC Melting Point Apparatus Model SMP3. The TLCs were carried out on Eastman Chromatogram Silica Gel Sheets (13,181; 6060) with fluorescent indicator. A mixture of hexane and ethyl acetate (1:2) was used as eluent and iodine was used as revelator for the chromatograms. The IR spectra were measured with a Fourier Transform Infrared spectrometer Brucker Alpha. The UV spectra were recorded with a JENWAY 6715 UV–Vis Spectrophotometer. Combustion analyses were carried out with a C, H, N, and S Euro EA from Hekatech Company, their results were found to be in good agreement (± 0.3%) with the calculated values. XRD data was collected on a STOE Stadi-p X-ray powder diffractometer (STOE & Cie GmbH, Darmstadt, Germany) with Cu K_α1_ radiation (λ = 1.54056 Å; Gemonochromator; flat samples) in transmission geometry with a DECTRIS® MYTHEN 1 K detector (DECTRIS, Baden-Daettwil, Switzerland). HR-ESI–MS spectra were performed with a spectrometer (QTOF Bruker, Germany) equipped with a HR-ESI source. The spectrometer operates in positive ion mode (mass range: 100–1500, with a scan rate of 1.00) with automatic gain control to provide high-accuracy mass measurements within 0.40 ppm deviation using Na formate as calibrant. The following parameters were used for experiments: spray voltage of 4.5 kV, capillary temperature of 200 °C. Nitrogen was used as sheath gas (10 l/ min). The spectrometer was attached to an Ultimate 3000 (Thermo Fisher, Germany) UHPLC system consisting of LC-pump, Diode Array Detector (DAD) (*λ*: 190–600 nm), auto-sampler (injection volume 10 μl) and column oven (40 °C). The separations were performed using a Synergi MAX-RP 100 A (50 × 2 mm, 2.5 μm particle size) with a H2O (+ 0.1% HCOOH) (A)/acetonitrile (+ 0.1% HCOOH) (B) gradient (flow rate.

500 μL/min, injection volume 5 μl). ^1^H NMR spectra and ^13^C NMR spectra were recorded in deuterated chloroform on a Bruker SF spectrometer operating respectively at 400 and 100 MHz; TMS was used as internal reference.

#### Synthesis of 9,10-dihydro-9,10-ethanoanthracene-11,12-dicarboxylic anhydride (3)

4.92 g (27.76 mmol) of anthracene and 2.43 g (23.80 mmol) of maleic anhydride are refluxed for 40 min in 50 ml of xylene. The solution obtained is filtered hot and left to stand for about a day for gentle and gradual crystallization of the product. The latter is then filtered, dried and crystallized from xylene to give 6.30 g (83%) of white crystals; mp: 315 °C (Lit. [25]: 261–262 °C from xylene).

#### Synthesis of 13-(4-hydroxyphenyl)-9,10-dihydro-9,10-ethanoanthracene-12,14-dicarboximide (5)

4 g (0.0145 mol) of **3** are dissolved in 50 ml of acetic acid while hot. Excess para-aminophenol (4 g, 0.0367 mol) previously dissolved in 30 ml of acetic acid is added and the mixture is heated under reflux for 3 h and then cooled to room temperature. The solution is filtered and washed with aqueous ethanol (50%) and dried to give 4.84 g of a gray-colored product **5**; mp: 337 °C (Lit [29]: 334–335 °C from DMF), yield 92% (Lit [29]: 57%); ESI–MS: 390.11 (M + Na, 100%). UV–Vis: λ_max_ (DMSO): 250, 355, 395 nm. IR (potassium bromide): 3363 cm^−1^ (OH), 2973 (CH), 1696 (C = O), 1600–1562 (C_Ar_ = C_Ar_), 1273 (C-N), 1202 (C-O), 764 (= C_Ar_H) cm^−1^. ^1^H NMR (CDCl_3_) *δ* 7.32 (dd, 2H, J = 5.3 and 3.3 Hz, H-3, H-7), 7. 23 (dd, 2H, J = 5.4 and 3.3 Hz, H-2, H-6), 7.11 (m, 4H, H- 1, H-4, H-5, H-8). 4.75 (s, 2H, H-9, H-10), 3.25 (s, 2H, H-11, H-15), 6.62 (d, 2H, J = 8.8 Hz, H-2′, H-6′), 6.17 (d, 2H, J = 8.8 Hz, H-3′, H-5′). ^13^C (^1^H)-NMR (CDCl_3_) *δ*177.9 (C-12, C-14), 157.4 (C-4′), 141.2 (C-4a, C-8a), 138.8 (C-1a, C-5a), 122.7 (C-1’), 127.6 (C-3′, C-5′), 127.1 (C-1, C-5), 126.8 (C-4, C-8), 125.1 (C-3, C-7), 124.3 (C-2, C-6), 115.9 (C-2′, C-6′), 46.9 (C-11, C-15) and 45.8 (C-9, C-10). Anal. Calcd. for C_24_H_17_NO_3_ (367.12): C, 78.56; H, 4.66; N, 3.8; found: C, 78.35; H, 4.83; N, 3.91.

#### Synthesis of 13-(4-hydroxy-3-(phenyldiazenyl) phenyl)-9,10-dihydro-9,10-ethanoanthracene-12,14–dicarboximide (8)

##### Preparation of diazonium salt solution

Dry sodium nitrite (1.38 g, 2 mmol) was slowly added over a period of 30 min to concentrated sulphuric acid (5 mL) with occasional stirring. The solution was cooled to 0–5 °C. 1 g (1.07 mmol) of aniline (**6**) was dissolved in DMSO (5 mL) and cooled to 0–5 °C. The nitrosyl sulphuric acid solution was added to the amine solutionand the temperature was maintained to 0–5 °C.

##### Procedure for the preparation of the coupling product

Compound **5** (0.367 g, 1 mmol) was dissolved in DMSO (5 mL) and then cooled in an ice-bath at 0–5 °C. The diazonium solution **7** previously prepared was added drop wise over 1 h before neutralizing the sulfuric acid present with a 10 ml sodium acetate (10%) solution. 50 ml of ice-cold water was then added and the solution was filtered off after 30 min and rinsed with iced water. After crystallization from ethanol After crystallization from ethanol (98%), 315 mg of compound **8** was obtained as brown powder; mp: 271 °C, Yield 67%; ESI–MS: 494.22 (M + Na, 100%). UV–Vis: λ_max_ (DMSO) = 385 nm; IR (potassium bromide): 3367 cm^−1^ (OH), 3060 (CH), 1696 (C = O), 1598 (C_Ar_ = C_Ar_), 1465 (-N = N-), 1274 (C-N), 1202 (C-O), 764 (= C_Ar_H) cm^−1^; ^1^H NMR(CDCl_3_) *δ* ppm: 7.45 (m, 5H, H-2″, H-3″, H-4″, H-5″, H-6″). 7.40 (dd, 2H, J = 5.3 and 3.2 Hz, H-3, H-7), 7.32 (dd, 2H, J = 5.3 and 3.3 Hz, H-2, H-6), 7.19 (m, 4H, H-1, H-4, H-5, H-8), 6.98 (s, 1H, H-6′), 6.68 (d, 1H, J = 8.1, H-2′), 6.18 (d, 1H, J = 8.2, H-3′), 4.75 (s, 2H, H-9, H-10), 3.25 (s, 2H, H-11, H-15), ^13^C NMR (CDCl_3_) *δ* ppm: 176.5, 160.7, 152.8, 141.4, 138.8, 130.8, 129.1, 127.8, 127.2, 126.9, 125.2, 124, 122.9, 116.0, 47.0 and 46.1. Anal. Calcd. for C_30_H_21_N_3_O_3_ (471.16): C, 76.42; H, 4.49; N, 8.91; found: C, 76.12; H, 4.68; N, 8.68.

### Antimicrobial evaluation

#### Tested microorganisms

The antimicrobial activity was performed against four bacterial and three fungal species. The selected microorganisms were one Gram-positive *Staphylococcus aureus* ATCC25923, three Gram-negative *Vibrio cholera* NB2, *V. cholera* SG24 and *V. cholera* CO6 and three yeast strains *Candida albicans* ATCC10231, *Candida tropicalis* PK233 and *Cryptococcus neoformans* H99. These microorganisms were taken from our laboratory collection. The fungal and bacterial strains were grown at 37 °C and maintained on Sabouraud Dextrose Agar (SDA, Conda, Madrid, Spain) and nutrient agar (NA, Conda) slants respectively.

#### Determination of Minimum Inhibitory Concentration (MIC) and Minimum Microbicidal Concentration (MMC)

The antibacterial and antifungal activity was evaluated by determining the MICs and MMCs as previously described [28]. MICs of synthesized compounds were determined by broth micro dilution. Each test sample was dissolved in dimethylsulfoxide (DMSO) to give a stock solution. This was serially diluted two-fold in Mueller–Hinton Broth (MHB) for bacteria and Sabouraud Dextrose Broth (SDB) for fungi to obtain concentration ranges of 512 to 0.25 μg/mL. Then, 100 µL of each sample concentration was added to respective wells (96-well micro plate) containing 90 µL of SDB/MHB and 10 µL of inoculum to give final concentration ranges of 256 to 0.125 μg/mL. The final concentrations of microbial suspensions were 2.5 × 10^5^ cells/mL for yeasts and 10^6^ CFU/mL for bacteria. Dilutions of nystatin (Sigma-Aldrich, Steinheim, Germany) and ciprofloxacin (Sigma-Aldrich, Steinheim, Germany) were used as positive controls for yeasts and bacteria respectively. Broth with 10 µL of DMSO was used as negative control. MICs were assessed visually and were taken as the lowest sample concentration at which there was no growth or virtually no growth. The lowest concentration that yielded no growth after the sub-culturing was considered as the MBCs or MFCs. All the tests were performed in triplicate [28].

#### Cytotoxicity assay

Whole blood (10 mL) from albino rats was collected by cardiac puncture in EDTA tubes. The study was conducted according to the institutional guidelines and approved by the Cameroon National Ethical Committee (Reg. No. FWA-IRB00001954) and in compliance with the ARRIVE guidelines. Erythrocytes were harvested by centrifugation at room temperature for 10 min at 1000 xg and were washed three times in PBS buffer [30].The cytotoxicity was performed as previously described [30].

## Supplementary Information

**Additional file 1: Figure S1.** UV Spectrum of compound **5**. **Figure S2.** IR Spectrum of compound **5**. **Figure S3.** Mass Spectrum of compound **5**. **Figure S4.** ^1^H NMR of compound **5**. **Figure S5.** COSY of compound **5**. **Figure S6.** ^13^C NMR of compound **5**. **Figure S7.** HSQCSpectrum of compound **5**. **Figure S8.** HMBC Spectrum of compound **5**. **Figure S9.** UV Spectrum of compound **8**. **Figure S10.** IR Spectrum of compound **8**. **Figure S11.** HRMS ESI-Positive mode of compound **8**. **Figure S12.** Mass Spectrumfragmentation of compound **8**. **Figure S13.** ^1^H NMR of compound **8**. **Figure S14.** ^13^C NMR of compound **8**. **Table S1.** ARRIVE Essential 10.** Table S2.** ARRIVE Recommended Set.

## Data Availability

All spectra for the compound characterization are provided as Additional file 1. Also, the raw data for all biological evaluations are available from the corresponding author upon reasonable request.

## Abbreviations

UV: Ultra-violet
IR: Infra-red
FTIR: Fourier-transform infrared spectroscopy
HRMS: High resolution mass spectroscopy
^1^H NMR: Proton nuclear magnetic resonance
^13^C NMR: Carbon nuclear magnetic resonance
µg: Microgram
°C: Degree centigrade
h: Hour
g: Gram
mg: Milligram
L: Liter
mL: Milliliter
µL: Microliter
MIC: Minimum Inhibitory Concentration
MMC: Minimum Microbicidal Concentration
DMSO: Dimethylsulfoxide
