# Supplementary material for: Synthesis, characterization and antimicrobial properties of two derivatives of pyrrolidine-2,5-dione fused at positions-3,4 to a dibenzobarrelene backbone
Source: BMC Chem. 2022 Mar 3;16(1):8. doi: 10.1186/s13065-022-00801-5 (PMC8895586; doi:10.1186/s13065-022-00801-5)
Supplement: Supplementary file 1 — Additional file 1: Figure S1. UV Spectrum of compound 5. Figure S2. IR Spectrum of compound 5. Figure S3. Mass Spectrum of compound 5. Figure S4. 1H NMR of compound 5. Figure S5. COSY of compound 5. Figure S6. 13C NMR of compound 5. Figure S7. HSQCSpectrum of compound 5. Figure S8. HMBC Spectrum of compound 5. Figure S9. UV Spectrum of compound 8. Figure S10. IR Spectrum of compound 8. Figure S11. HRMS ESI-Positive mode of compound 8. Figure S12. Mass Spectrumfragmentation of compound 8. Figure S13. 1H NMR of compound 8. Figure S14. 13C NMR of compound 8. Table S1. ARRIVE Essential 10. Table S2. ARRIVE Recommended Set. [file 13065_2022_801_MOESM1_ESM.doc]

**SUPPLEMENTARY MATERIALS**

**Synthesis, characterization and antimicrobial properties of two derivatives of pyrrolidine-2,5-dione fused at positions-3,4 to a dibenzobarrelene backbone**

Emmanuel Sopbué Fondjo^1^*, NjoyaAbdou Salamou^1^, Jean-de-Dieu Tamokou^2^, Giscard Doungmo^3^, Bruno Djakou Lenta^4^, Peter F. W. Simon^5^, Appolinaire Tsopmo^6^, Jules-Roger Kuiate^2^

^1^*Laboratory of Applied Synthetic Organic Chemistry, Department of Chemistry, Faculty of Science, University of Dschang, P.O. Box 67 Dschang, Republic of Cameroon.* ^2^*Research Unit of Microbiology and antimicrobial Substances, Department of Biochemistry, Faculty of Science, University of Dschang, PO Box 067 Dschang, Republic of Cameroon. ^3^Institut fürAnorganischeChemie, Christian-Albrechts-Universitätzu Kiel, Max-Eyth-Str. 2, 24118 Kiel, Germany. ^4^Higher Teacher’s Training College, University of Yaounde I; P. O. Box 47, Yaounde, Cameroon.* ^5^*Polymer Chemistry Laboratory, Faculty of Live Sciences, Rhine-Waal University of Applied Sciences, Campus Kleve, Marie-Curie Strasse 1, D-47533 Kleve, Germany.* ^6^*Department of Chemistry, Carleton University, 1125 Colonel By DriveK1S 5B6, Ottawa, Canada+1-613-520-260 Ext 3122.*

Table of contents

| Figures | Materials | Pages |
| --- | --- | --- |
| Figure 1 | UV spectrum of compound **5** in ethanol as solvent………………………………….. | 2 |
| Figure 2 | IR spectrum of compound **5**…………………………………………………………………….. | 2 |
| Figure 3 | HRMS ESI-Positive mode of compound **5**………………………………………………… | 3 |
| Figure 4 | ^1^H-NMR (CDCl_3_, 400 MHz) spectrum of compound **5**……………………………….. | 3 |
| Figure 5 | COSY Spectrum of compound**5**……………………………………………………………….. | 4 |
| Figure 6 | ^13^C-NMR (CDCl_3_, 100 MHz) Spectrum of**5** ……………………………………… | 5 |
| Figure 7 | HSQC (CDCl_3_, 100 MHz) spectrum of **5**……………………………………………………. | 5 |
| Figure 8 | HMBC (CDCl_3_, 100 MHz) spectrum of **5**……………..…………………………………….. | 6 |
| Figure 9 | UV spectrum of compound **8**inethanol as solvent …………………………………. | 6 |
| Figure 10 | IR spectrum of compound **8**…………………..………………………………………………… | 7 |
| Figure 11 | HRMS ESI-Positive mode of compound **8** ……………….…………….…………………. | 7 |
| Figure 12 | Mass Spectrum fragmentation of compound **8**…………………………………………. | 8 |
| Figure 13 | ^1^H-NMR (CDCl_3_, 400 MHz) spectrum of compound **8**……………….……............ | 8 |
| Figure 14 | ^13^C-NMR (CDCl_3_, 100 MHz) Spectrum ofcompound **8**……………….…….......... | 9 |

Compound 5

**Figure S1:**UV Spectrum of compound **5**

OH

C=O

C=C

=CH

C-N

C-O

=CH

**Figure S2:**IR Spectrum of compound **5**

[M+Na]^+^

[2M+Na]^+^

**Figure S3:** Mass Spectrum of compound**5**


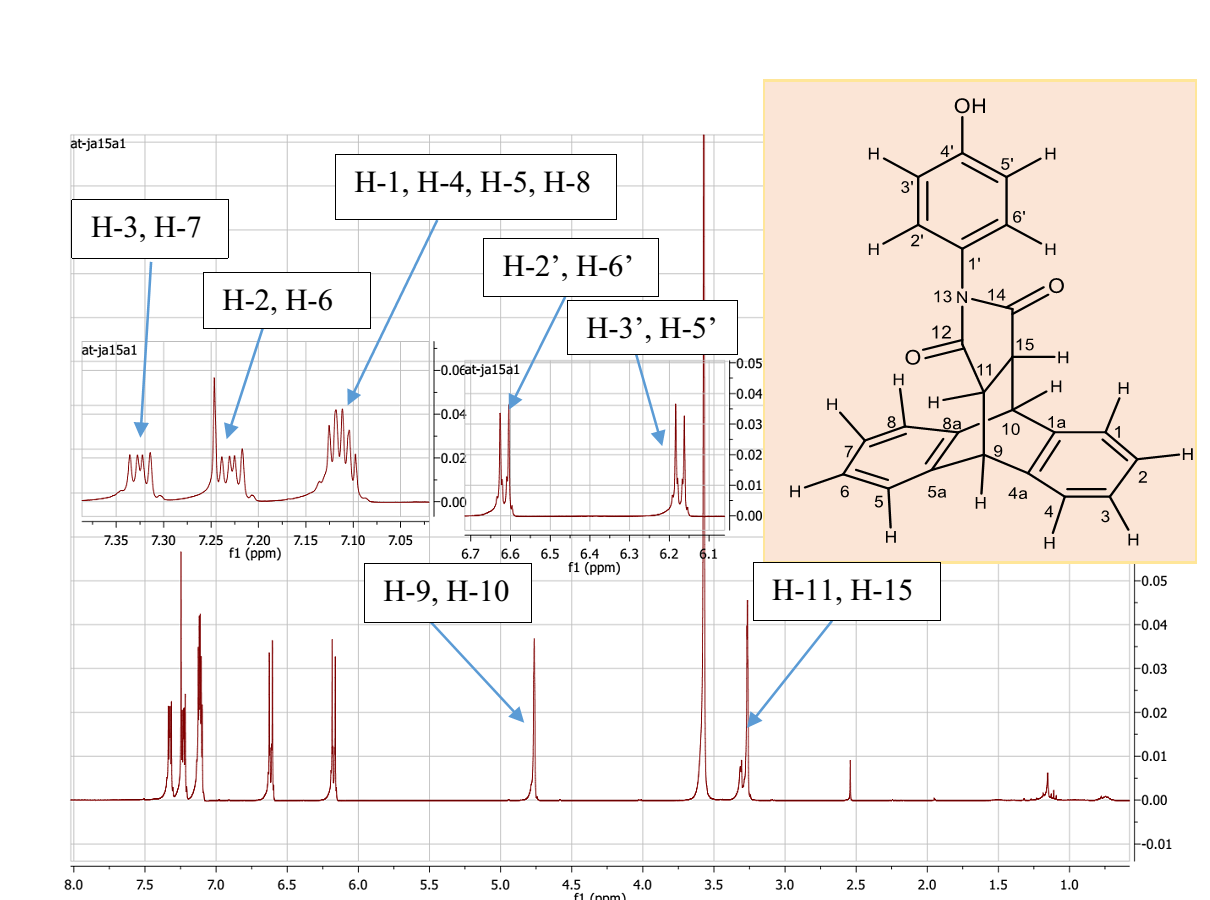


**Figure S4 :**^1^H NMR of compound **5**


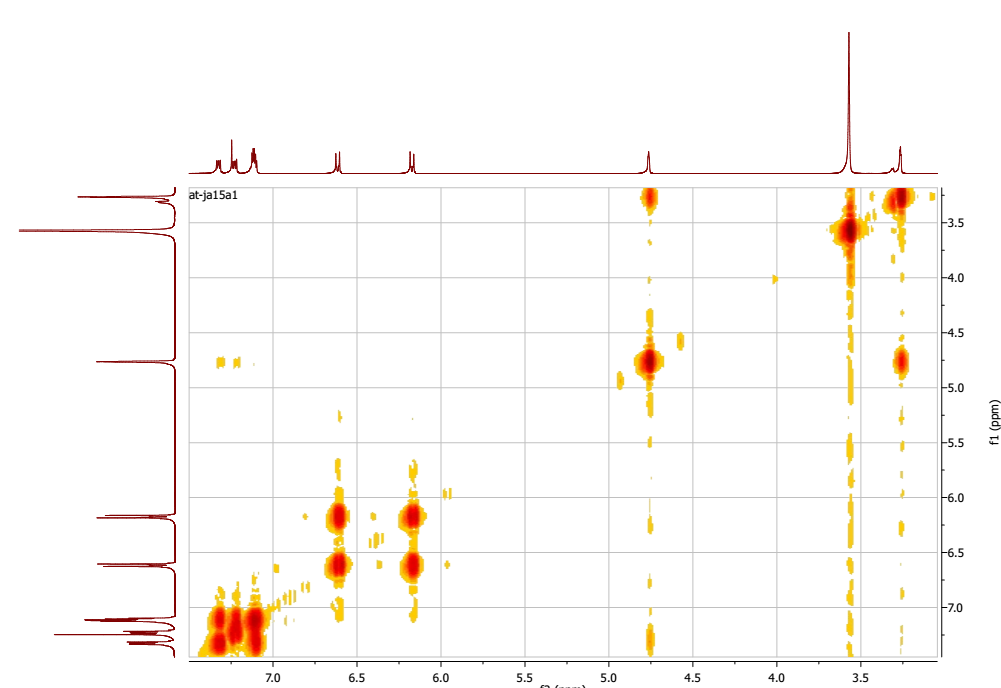


H-2’

H-3’

H-3 H-1

H-2

H-1

H-9

H-11

H-11

H-9

H-3’

H-2’

**Figure S5 :**COSY of compound **5**


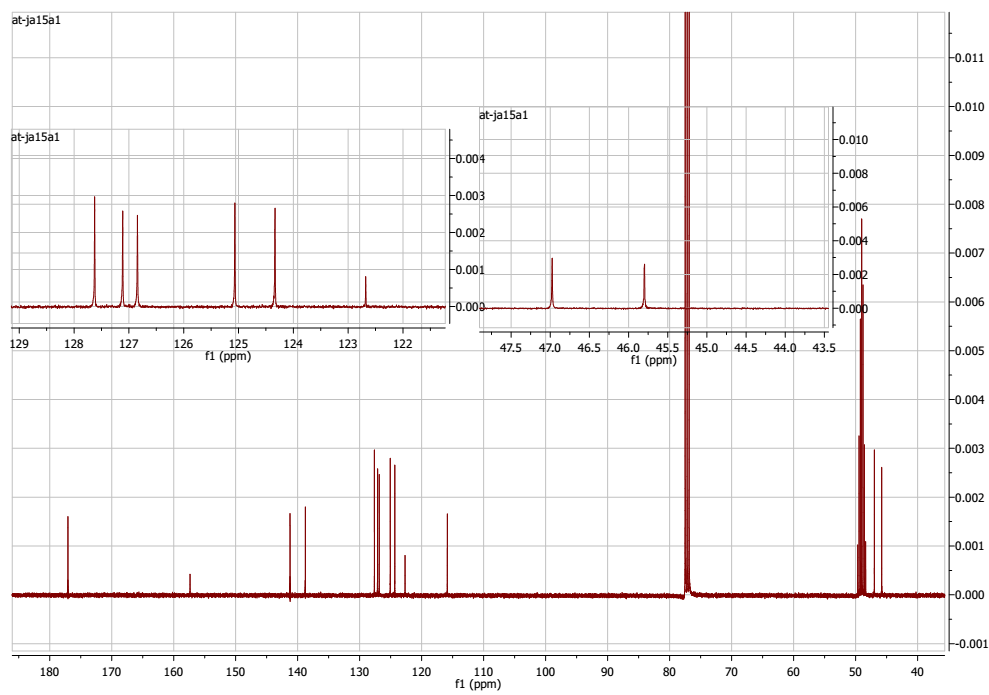


C-12, C-14

C-4’

C-3’, C-5’

C-1, C-5

C-11, C-15

C-9, C-10

C-2’, C-6’

C-4a, C-8a

C-1a, C-5a

C-3, C-7

C-1’

C-4, C-8

C-2,C-6

**Figure S6 :** ^13^C NMR of compound **5**

H-1,4


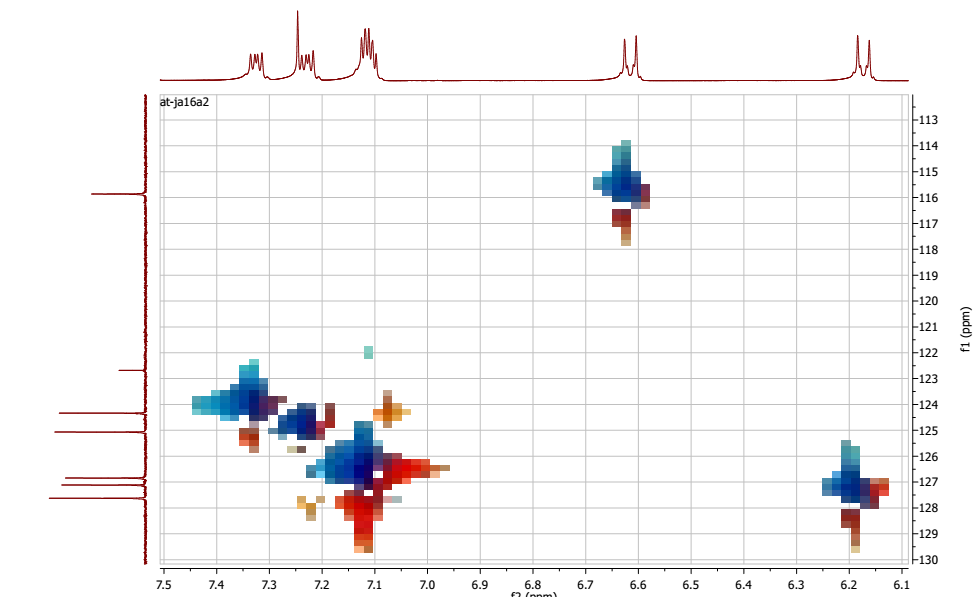


H-2’

H-3’

H-2

H-3

C-2’

C-2

C-3

C-3’

C-1,4

**Figure S7 :** HSQCSpectrum of compound **5**


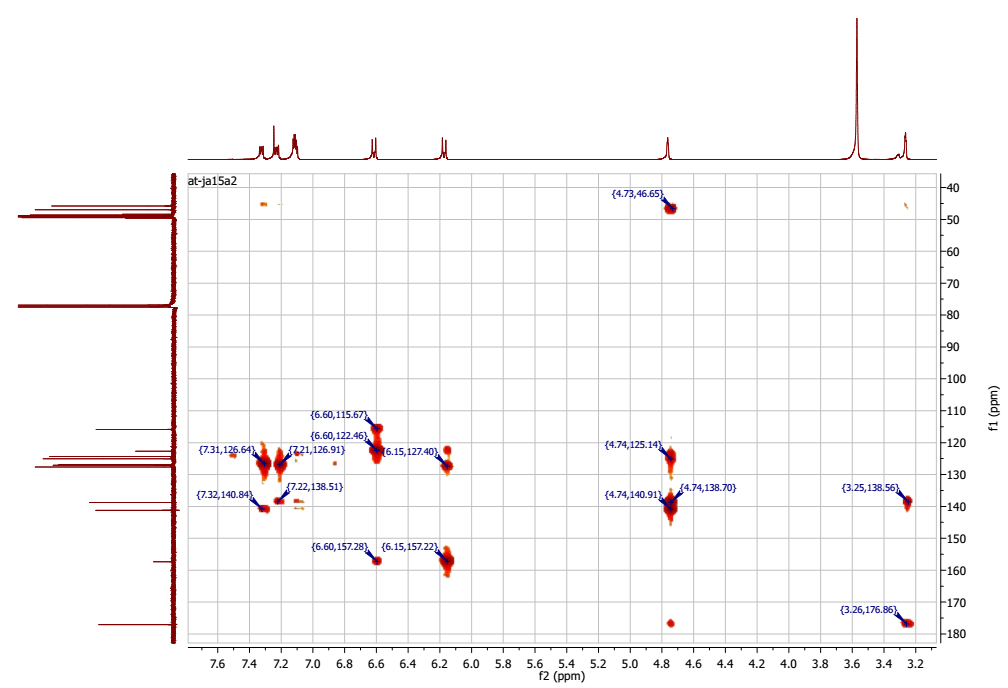


**Figure S8** : HMBC Spectrum of compound **5**

Compound 8

**Figure S9 :**UV Spectrum of compound **8**


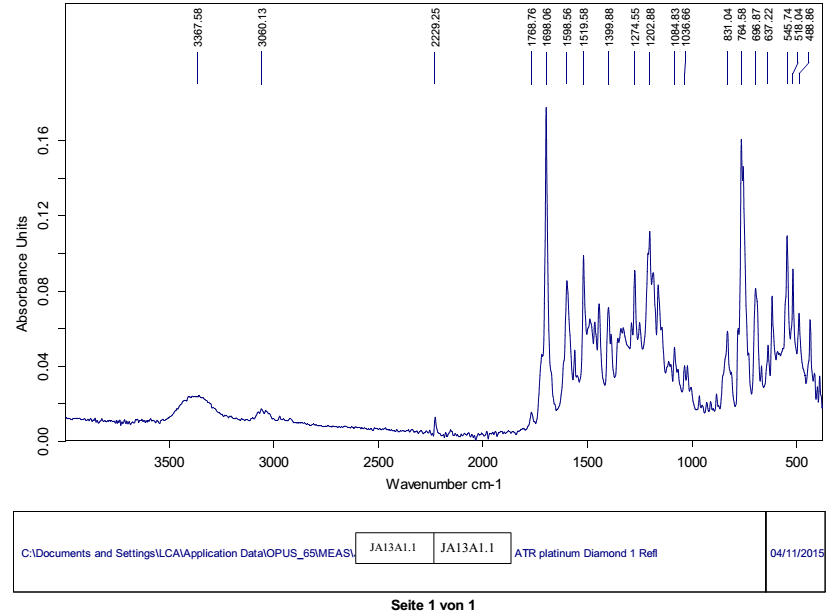


OH

=CH

=CH

C=C

C=O

N=N

C-O

C-N

N-H

**Figure S10 :**  IR Spectrum of compound **8**

[M+Na]^+^

**Figure S11 :** HRMS ESI-Positive mode of compound **8**

**Figure S12 :** Mass Spectrumfragmentation of compound **8**


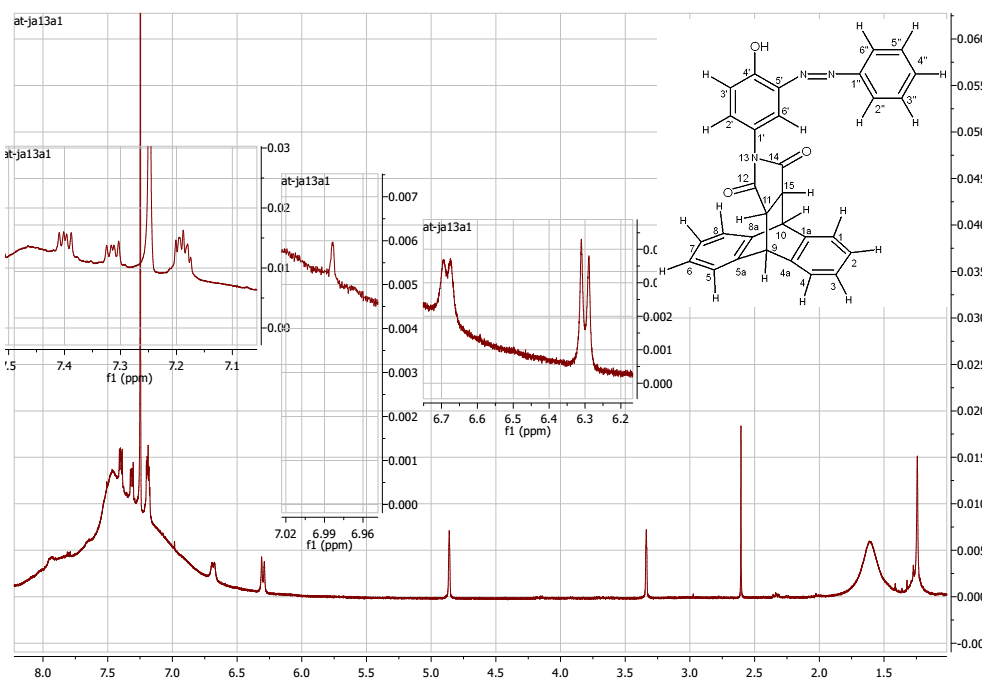


H-2, H-6

H-3, H-7

H-1, H-4, H-5, H-8

H-6’

H-2’

H-3’ H-7

H-2’’, H-3’’,H-4’’, H-5’’, H-6’’

H_OH_

**Figure S13 :**^1^H NMR of compound **8**


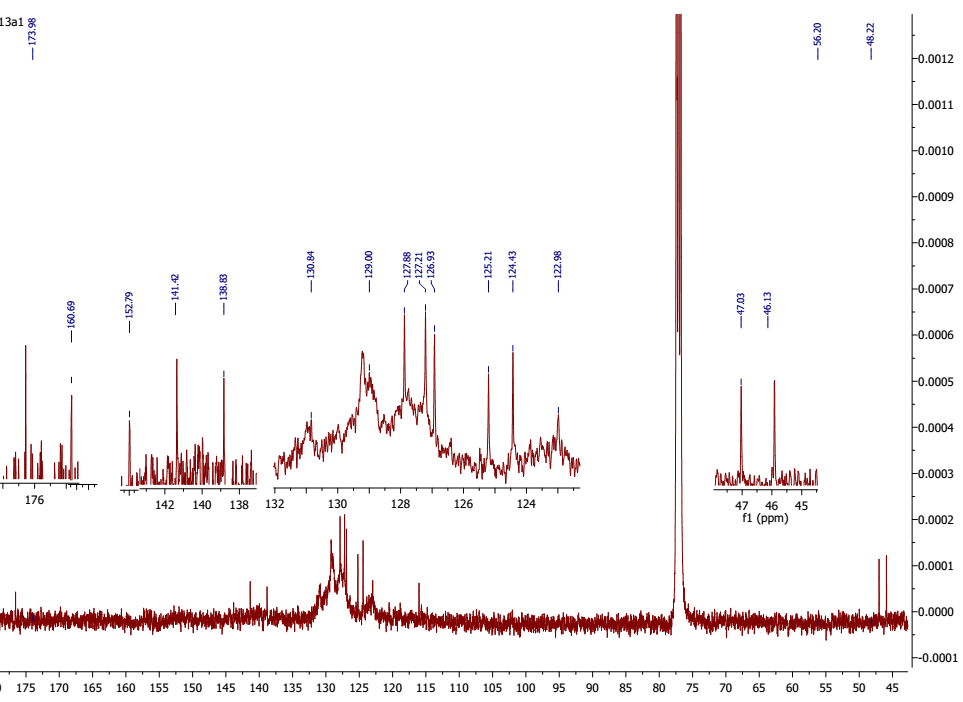


C-3’

C-1’’

C-12, C-14

C-4’

C-1’

C-11, C-15

C-2’’, C-3’’, C-4’’, C-5’’, C-6’’

C-5’

C-9, C-10

C-2’, C-6’

C-4a, C-8a

C-1a, C-5a

C-2,C-6

C-1, C-5

C-4, C-8

C-3, C-7

**Figure S14 :**^13^C NMR of compound**8**

**[Table S1.](../../../../C:%5CUsers%5CFREDER~1%5CAppData%5CLocal%5CTemp%5CARRIVE%20guidelines%20pbio.3000410%202021.doc#page3)  ARRIVE Essential 10.**

|  |  |  |  | **ARRIVE Essential 10** |  |
| --- | --- | --- | --- | --- | --- |
|  |  |  |  |  |  |
| Study design | 1 | |  | For each experiment, provide brief details of study design including: |  |
|  |  |  |  | a. The groups being compared, including control groups. If no control group hasbeen used, the rationale should be stated. | N/A |
|  |  |  |  |
|  |  |  |  | b. The experimental unit (e.g., a single animal, litter, or cage of animals). | Methods, Cytotoxicity assay |
|  |  |  |  |  |  |
| Sample size | 2 | |  | a. Specify the exact number of experimental units allocated to each group, and thetotal number in each experiment. Also indicate the total number of animals used. | Methods, Cytotoxicity assay |
|  |  |  |  |
|  |  |  |  | b. Explain how the sample size was decided. Provide details of any a priori samplesize calculation, if done. | N/A |
|  |  |  |  |
|  |  |  |  |  |  |
| Inclusion and exclusion  criteria | 3 | |  | a. Describe any criteria used for including and excluding animals (or experimentalunits) during the experiment, and data points during the analysis. Specify if thesecriteria were established a priori. If no criteria were set, state this explicitly. | N/A |
|  |  |  |
|  |  |  |  | b. For each experimental group, report any animals, experimental units, or datapoints not included in the analysis and explain why. If there were no exclusions,state so. | N/A |
|  |  |  |  |
|  |  |  |  |
|  |  |  |  | c. For each analysis, report the exact value of *n* in each experimental group. | N/A |
| Randomisation | 4 | |  | a. State whether randomisation was used to allocate experimental units to controland treatment groups. If done, provide the method used to generate therandomisation sequence. | N/A |
|  |  |  |  |
|  |  |  |  |
|  |  |  |  | b. Describe the strategy used to minimise potential confounders such as the orderof treatments and measurements, or animal/cage location. If confounders were notcontrolled, state this explicitly. | N/A |
|  |  |  |  |
|  |  |  |  |
|  |  |  |  |  |  |
| Blinding | 5 | |  | Describe who was aware of the group allocation at the different stages of theexperiment (during the allocation, the conduct of the experiment, the outcomeassessment, and the data analysis). | N/A |
|  |  |  |  |
|  |  |  |  |
|  |  |  |  |  |  |
| Outcomemeasures | 6 | |  | a. Clearly define all outcome measures assessed (e.g., cell death, molecular markers,or behavioural changes). | Methods, Cytotoxicity assay |
|  |  |  |  |
|  |  |  |  | b. For hypothesis-testing studies, specify the primary outcome measure, i.e., theoutcome measure that was used to determine the sample size. | N/A |
|  |  |  |  |
|  |  |  |  |  |  |
| Statisticalmethods | 7 | |  | a. Provide details of the statistical methods used for each analysis, includingsoftware used. | N/A |
|  |  |  |  |
|  |  |  |  | b. Describe any methods used to assess whether the data met the assumptions of thestatistical approach, and what was done if the assumptions were not met. | N/A |
|  |  |  |  |
|  |  |  |  |  |  |
| Experimentalanimals | 8 | |  | a. Provide species-appropriate details of the animals used, including species, strainand substrain, sex, age or developmental stage, and, if relevant, weight. | Methods, Cytotoxicity assay, |
|  |  |  |  |
|  |  |  |  | b. Provide further relevant information on the provenance of animals, health/immune status, genetic modification status, genotype, and any previousprocedures. | Methods, Cytotoxicity assay |
|  |  |  |  |
|  |  |  |  |
|  |  |  |  |  |  |
| Experimentalprocedures | 9 | |  | For each experimental group, including controls, describe the procedures inenough detail to allow others to replicate them, including: |  |
|  |  |  |  | a. What was done, how it was done, and what was used. | N/A |
|  |  |  |  | b. When and how often. | N/A |
|  |  |  |  | c. Where (including detail of any acclimatisation periods). | N/A |
|  |  |  |  | d. Why (provide rationale for procedures). | N/A |
|  |  |  |  |  |  |
| Results | 10 | |  | For each experiment conducted, including independent replications, report: |  |
|  |  |  |  | a. Summary/descriptive statistics for each experimental group, with a measure of  variability where applicable (e.g., mean and SD, or median and range).  b. If applicable, the effect size with a confidence interval. | N/A  N/A |

[**Table S2.**](#_bookmark13) **ARRIVE Recommended Set**

| **Recommended Set** | | |  |
| --- | --- | --- | --- |
| Abstract | 11 | Provide an accurate summary of the research objectives, animal species, strain and sex, key methods, principal findings, and study conclusions. | N/A |
| Background | 12 | 1. Include sufficient scientific background to understand the rationale and context for the study, and explain the experimentalapproach. 2. Explainhowtheanimalspeciesandmodelusedaddressthescientific   objectives and, where appropriate, the relevance to human biology. | 1. N/A |
| Objectives | 13 | Clearly describe the research question, research objectives and, where appropriate, specific hypotheses being tested. | N/A |
| Ethical statement | 14 | Provide the name of the ethical review committee or equivalent that has approved the use of animals in this study, and any relevant licence or protocol numbers (if applicable). If ethical approval was not sought or granted, provide a  justification. | Methods, Cytotoxicity assay |
| Housing and husbandry | 15 | Provide details of housing and husbandry conditions, including any environmental enrichment. | Methods, Cytotoxicity assay |
| Animal care and monitoring | 16 | 1. Describe any interventions or steps taken in the experimental protocols to reduce pain, suffering, anddistress. 2. Report any expected or unexpected adverseevents. 3. Describe the humane endpoints established for the study, the signs thatweremonitored, and the frequency of monitoring. If the study did not have humane endpoints, state this. | 1. Methods, Cytotoxicity assay |
| Interpretation/scientific implications | 17 | 1. Interpret the results, taking into account the study objectives and hypotheses, current theory, and other relevant studies in theliterature. 2. Comment on the study limitations, including potential sources ofbias,   limitations of the animal model, and imprecision associated with the results. | 1. N/A |
| Generalisability/translation | 18 | Comment on whether, and how, the findings of this study are likely to  generalise to other species or experimental conditions, including any relevance to human biology (where appropriate). | N/A |
| Protocol registration | 19 | Provide a statement indicating whether a protocol (including the research question, key design features, and analysis plan) was prepared before the study,and if and where this protocol was registered. | N/A |
| Data access | 20 | Provide a statement describing if and where study data are available. | Declarations, Availability of data and materials |
| Declaration of interests | 21 | 1. Declare any potential conflicts of interest, including financial and nonfinancial. If none exist, this should bestated. 2. Listallfundingsources(includinggrantidentifier)andtheroleofthe   funder(s) in the design, analysis, and reporting of the study. | Declarations |
